# APOBEC3-mediated mutagenesis in cancer: causes, clinical significance and therapeutic potential

**DOI:** 10.1186/s13045-023-01425-5

**Published:** 2023-03-28

**Authors:** Kelly Butler, A. Rouf Banday

**Affiliations:** Genitourinary Malignancies Branch, Center for Cancer Research, National Cancer Institute, National Institutes of Health, Bethesda, MD 20892 USA

**Keywords:** APOBECs, Cancer, Somatic mutations, Germline genetics, Tumor microenvironment, Biomarkers, Immunotherapy

## Abstract

Apolipoprotein B mRNA-editing enzyme, catalytic polypeptides (APOBECs) are cytosine deaminases involved in innate and adaptive immunity. However, some APOBEC family members can also deaminate host genomes to generate oncogenic mutations. The resulting mutations, primarily signatures 2 and 13, occur in many tumor types and are among the most common mutational signatures in cancer. This review summarizes the current evidence implicating APOBEC3s as major mutators and outlines the exogenous and endogenous triggers of APOBEC3 expression and mutational activity. The review also discusses how APOBEC3-mediated mutagenesis impacts tumor evolution through both mutagenic and non-mutagenic pathways, including by inducing driver mutations and modulating the tumor immune microenvironment. Moving from molecular biology to clinical outcomes, the review concludes by summarizing the divergent prognostic significance of APOBEC3s across cancer types and their therapeutic potential in the current and future clinical landscapes.

## Background

Apolipoprotein B mRNA-editing enzyme, catalytic polypeptides (APOBECs) are a class of cytosine deaminases with eleven primary family members: APOBEC1, Activation-Induced Deaminase (AID), APOBEC2, APOBEC3 (A–H), and APOBEC4. Alternative splicing of APOBEC3B, APOBEC3H, and APOBEC3F further diversifies the APOBEC superfamily [1–4]. While all APOBEC family members share a conserved catalytic domain, they have distinct functions, mutational substrates, and tissue expression patterns [5]. AID, for example, is expressed in activated B cells and facilitates antibody diversification by deaminating immunoglobulin genes [6]. In contrast, APOBEC1 is expressed in the small intestine and edits mRNA to enable tissue-specific expression of a truncated but functionally important gastrointestinal protein [7–9]. APOBEC3s are much more widely expressed across human tissues and deaminate—and thereby damage—viral genomes as part of the innate immune response [10].

**Fig. 1:**
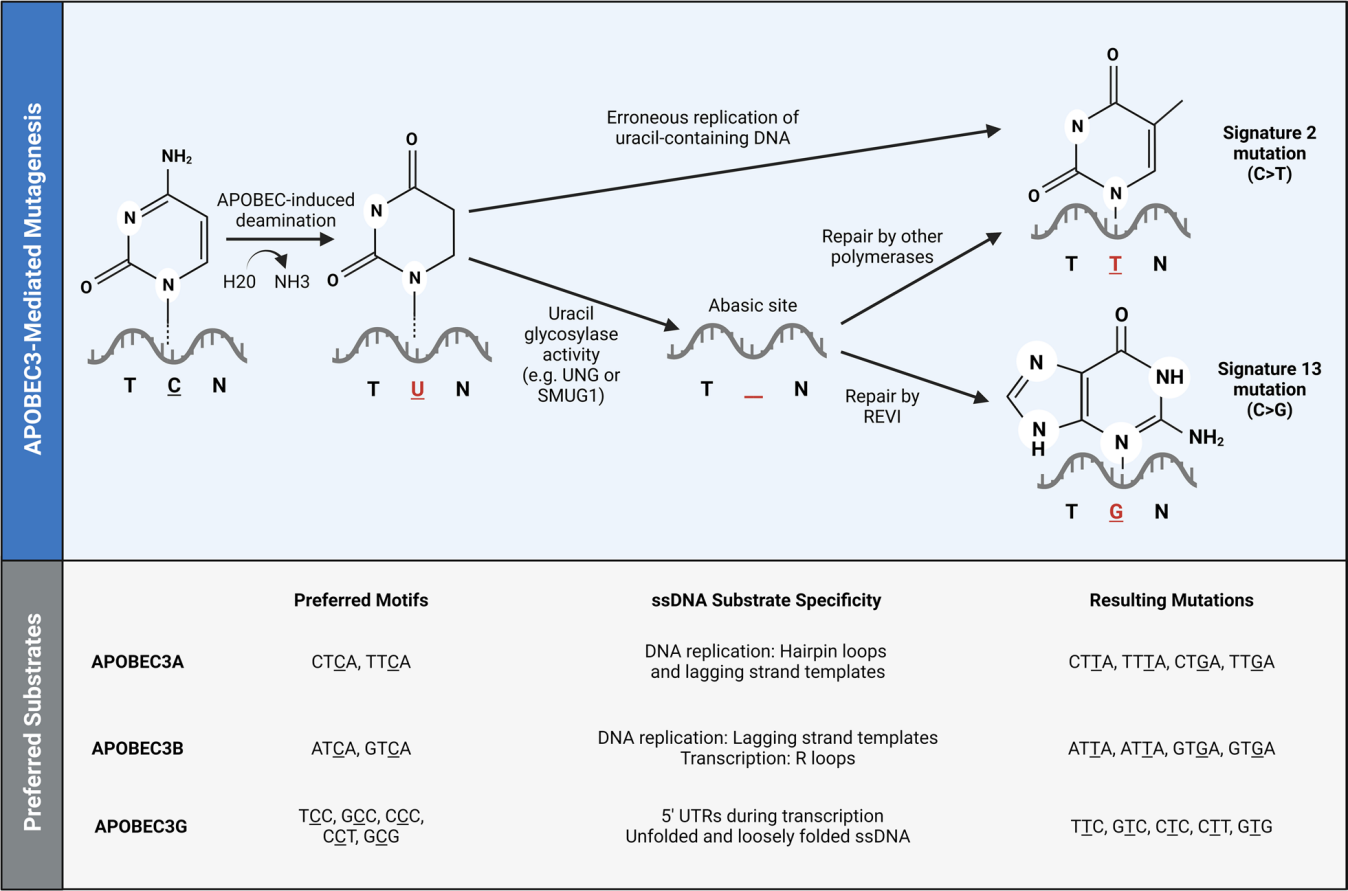
Mechanisms and preferred substrates for APOBEC3-mediated mutagenesis. Upper panel: APOBEC3s deaminate ssDNA, leaving uracil in the DNA template. Erroneous replication and repair pathways can then generate mutational signatures 2 and 13. Repair by the translesion synthesis (TLS) polymerase REVI generates a C-to-G mutation (signature 13), while repair by other enzymes such as DNA polymerase δ, DNA polymerase ε, and TLS polymerase κ generates a C-to-T mutation (signature 2) [20]. Lower panel: The major mutators among the APOBEC3 superfamily have distinct substrate preferences defined mainly by trinucleotide context and ssDNA secondary structure

Although APOBEC3s protect cells from viral infection, they also make host DNA vulnerable to mutations. APOBEC3-mediated mutagenesis begins with cytosine deamination, and all APOBEC3s can deaminate single-stranded DNA (ssDNA) with varying levels of enzymatic activity [11–13]. ssDNA substrates for APOBEC3s can transiently arise in the double-stranded genome during normal cellular processes such as DNA replication, transcription, and genomic repair. For example, both APOBEC3A and APOBEC3B can deaminate lagging strand templates during DNA replication [14–16]. APOBEC3A can also act on hairpin loops that form during DNA replication, while APOBEC3B preferentially deaminates R loops during transcription [17, 18]. APOBEC3G may similarly act on ssDNA during transcription, especially within 5′ UTRs [15]. Additionally, APOBEC3G has been shown to deaminate unfolded and loosely folded ssDNA (Fig. 1) [19].

Within ssDNA, the various APOEBC3s deaminate cytosines in distinct trinucleotide contexts. For example, APOBEC3A and APOBEC3B—which are the major mutators—deaminate thiamine preceding cytosine (TpC) motifs; APOBEC3A preferentially acts on TpC motifs following pyrimidines, while APOBEC3B tends to deaminate TpC motifs after purines [20–23]. Following deamination, different cellular processes can create C-to-T and C-to-G mutations, which are defined as signatures 2 and 13 in COSMIC, respectively [24–26]. The former C-to-T transition is more common and arises from aberrant replication of uracil-containing DNA templates, while both substitutions can occur through erroneous repair of abasic sites generated by uracil glycosylase activity (Fig. 1) [20, 27–29]. In addition to these conventionally defined APOBEC3-induced mutational signatures, APOBEC3G can cause C-to-T transitions at TCC, GCC, CCC, CCT, and GCG motifs (Fig. 1) [30].

APOBEC3-induced mutations are ubiquitous in cancer and may occur dispersed throughout the genome or in clusters. Over 75% of kataegis in tumor genomes have been attributed to APOBEC3 activity, compared to 15% of more diffuse omikli hypermutation [31, 32]. Overall, APOBEC3-induced mutations can constitute up to 68% of tumor mutation burden and are found in over half of all tumors; Only age-related signatures are more common [26, 27, 33].

Many of these APOBEC3-induced alterations are highly recurrent driver mutations affecting oncogenes and tumor suppressors, and APOBEC3s can also impact disease course through non-mutagenic pathways such as immune modulation in the tumor microenvironment. APOBEC3s can promote immune-activated or immunosuppressed phenotypes, which may partially explain their varying prognostic significance across cancer types. Based on clinical associations and pre-clinical studies, APOBEC3s could be used as biomarkers and targeted for therapies. The causes and clinical implications of APOBEC3-mediated mutagenesis are thus important areas of research and the focus of this review.

### Expression of APOBEC3s in cancers

APOBEC3s are expressed at low levels in many healthy tissues but are often overexpressed in tumors. Most studies have used RNA-based profiling to detect *APOBEC3* expression, and protein-based analyses have been more limited (Table 1). *APOBEC3B* is generally expressed at higher levels than other APOBEC3 family members, and an analysis of multiple cancers detected enrichment of *APOBEC3B* in eight tumor types: bladder, breast, head and neck, lung adenocarcinoma, lung squamous cell carcinoma, prostate, clear cell renal, and uterine [34]. High *APOBEC3B* expression was also observed in cervical and skin cancers, although healthy tissue was not available for comparison [34]. Similarly, high *APOBEC3B* levels have been reported in bladder, bile duct, lung, gastric, esophageal, neuroendocrine, and ovarian tumors [35–43].

**Table 1:** Overexpression and correlation of APOBEC3s with mutation burden across cancers

Cancer type	APOBEC3(s) overexpressed*	Expression type*	Correlated tumor mutation burden(s)**	References
Multiple	3A, 3B*, 3F, and 3G	mRNA*	APOBEC3-induced	[27]
Adrenal	3B	mRNA* and protein	Overall	[44]
B-cell lymphoma	3B and 3C	mRNA	N/A	[45]
Bile duct	3A* and 3B (No healthy tissue control)	mRNA	APOBEC3-induced	[43]
Bladder	3B*	Protein	N/A	[35]
	3B	mRNA	Not detected	[27]
	3B	mRNA	N/A	[34]
	3A* and 3B*	mRNA*	APOBEC3-induced	[1, 46]
	3B1	mRNA and protein	N/A	[1]
Breast	3A*, 3B*, and 3H	mRNA*	APOBEC3-induced	[27]
	3B	mRNA	N/A	[34]
	3B*	mRNA*	APOBEC3-induced, overall	[22]
	3A* and 3B	mRNA*	APOBEC-induced	[47]
	3A*, 3B*, and C3-H	mRNA*	APOBEC3-induced, overall	[48]
Cervical	3B	mRNA	N/A	[34]
Esophageal	3B*	mRNA and protein*	APOBEC3-induced in *PIK3CA*	[39]
Gastric	3B*	Protein*	Overall	[37]
	3B	mRNA and protein	N/A	[38]
Glioma	3B, 3C, 3D, 3F, 3G, 3H	mRNA*	Overall	[51]
Head and neck	3B	mRNA	N/A	[34]
Kidney	3B	mRNA	N/A	[34]
Leukemia	3A	mRNA	N/A	[52]
Lung	3B*	mRNA*	APOBEC3-induced	[27]
	3B*	mRNA*	APOBEC3-induced, overall	[53]
	3B*	mRNA*	APOBEC3-induced	[54]
	3B	mRNA	N/A	[34]
	3B	mRNA	N/A	[36]
Nasopharyngeal	3B	Protein	N/A	[55]
Ovarian	3B	Protein	N/A	[42]
	3B	mRNA* and protein	Overall	[41]
	3B*	mRNA*	Overall	[56]
Pancreatic	3G	mRNA and protein	N/A	[49]
Penile	3A (In subset)	Protein	N/A	[57]
Prostate	3B	mRNA	N/A	[34]
Skin	3B	mRNA	N/A	[34]
Uterus	3B	mRNA	N/A	[34]

^*^Indicates that a correlation was detected between the specified APOBEC3(s) and mutation burden(s). Some papers with both protein and mRNA expression only tested for correlations with one expression metric, so lack of an asterisk may indicate no relevant data rather than lack of statistical association

^**^N/A indicates no relevant data, not lack of statistical association

The expression of other APOBEC3s may also be dysregulated in cancer. *APOBEC3G*, for example, has been found at high levels in colon and pancreatic tumors [49, 50]. In breast cancer, studies have detected enrichment of *APOBEC3A*, *APOBEC3B*, and *APOBEC3H* [27, 48]. High *APOBEC3* expression has also been observed in multiple hematologic cancers. For example, *APOBEC3A* enrichment has been noted in leukemia, and both *APOBEC3B* and *APOBEC3C* have been found at elevated levels in primary effusion lymphoma [45, 52].

### APOBEC3A and APOBEC3B as the major mutators

APOBEC3 overexpression likely promotes mutagenesis, as multiple studies have identified correlations between APOBEC3 expression and a signature-specific mutation burden (Table 1). In a combined analysis of multiple tumor types, *APOBEC3B* expression was strongly associated with a higher APOBEC3-induced mutation load; *APOBEC3A*, *APOBEC3F*, and *APOBEC3G* showed similar but weaker correlations [27]. High *APOBEC3B* expression was also associated with more APOBEC3-induced mutations in lung cancer, while both *APOBEC3A* and *APOBEC3B* levels were correlated with APOBEC3-induced mutations in breast cancer [22, 47, 53, 54]. Similar associations have been observed in bladder cancer, which has one of the highest APOBEC3-induced mutation burden [1, 27, 34, 46]. In cholangiocarcinoma, only *APOBEC3A* expression was associated with APOBEC3-induced mutation burden [43].

These correlations suggest that APOBEC3A and APOBEC3B both contribute to mutagenesis, but the relative importance of these family members remains controversial [47, 58]. APOBEC3B is often assumed to be the major mutator given its higher expression in many tumors [22, 26, 34–36, 45, 48, 59]. However, APOBEC3A has greater enzymatic activity, which may allow it to generate more mutations despite generally lower tissue expression [47, 60]. Accordingly, comparison of *APOBEC3* knockout cell lines found that APOBEC3A deficiency has largest effect on mutagenesis [58]. This result corroborates earlier findings in yeast, which first distinguished APOBEC3A- and APOBEC3B-induced mutations and found that the former are more abundant in tumor genomes [23]. Further implicating APOBEC3A as a predominant mutator, an *APOBEC3B* germline deletion that generates a chimera of the *APOBEC3A* coding region fused to the 5′ UTR of *APOBEC3B* has been associated with more APOBEC-induced mutations in some cancers [61–64].

Other APOBEC3 family members likely induce mutations, as an in vitro analysis detected continued—though significantly decreased—acquisition of signatures 2 and 13 despite knocking out both *APOBEC3A* and *APOBEC3B* [58]. APOBEC3H may contribute to this residual mutagenesis, especially in cancers with APOBEC3H haplotype I, which has strong enzymatic activity and increased nuclear localization [65]. APOBEC3G could also be mutagenic since its expression has been associated with a distinct mutational signature [30]. Multiple APOBEC3s may thus induce mutations in cancer, with the most significant mutators potentially varying across tumors.

## Exogenous and endogenous triggers of APOBEC3-mediated mutagenesis

### Viral infection

APOBEC3s are interferon-stimulated genes induced by a wide variety of viruses, including polyomaviruses, parvoviruses, herpesviruses, and hepatitis B viruses [66]. Many virus-associated cancers thus have a high loads of mutation signatures 2 and 13. Cervical cancer, for example, is caused by human papillomavirus (HPV) in over 95% of cases and has abundant APOBEC3-induced mutations [67, 68]. APOBEC-induced mutations are also common in head and neck squamous cell carcinoma (HNSCC), with a strong correlation between HPV positivity and mutation signatures 2 and 13 [69].

Viral infection may also contribute to APOBEC3-mediated mutagenesis in some cancers that are not traditionally understood as virus-associated. According to the “hit and run” hypothesis, viral infection can trigger APOBEC3 activity early in tumorigenesis but is cleared before tumor detection [70]. This postulation could be relevant for some cases of bladder cancer, as a history of BK polyomavirus (BKPyV)-positive urine has been associated with increased bladder cancer risk [71]. BKPyV infection was also shown to induce APOBEC3 expression and deamination activity in an in vitro model of the normal human urothelium [72]. The potential risk of BKPyV-induced bladder carcinogenesis may be especially high in immunocompromised populations, specifically in organ transplant recipients. Accordingly, BKPyV viremia or other polyomavirus-related complications have been associated with a four-fold higher risk of bladder cancer following kidney transplantation [73–77]. Deep sequencing of bladder tumor genomes from organ transplant recipients has also revealed BKPyV integration [78–80].

While certain viral infections may contribute to APOBEC3-mediated mutagenesis in some traditionally non-viral cancers, additional factors are likely important given the continued acquisition of APOBEC3-induced mutations late in tumor evolution and presumably after infection clearance [22, 56, 81, 82]. Such non-viral factors may also explain the prevalence of APOBEC3-induced mutations in cancers for which hit-and-run viral etiology is less plausible.

### Inflammation

A myriad of factors can induce inflammation, which may increase APOBEC3 expression via NF-κB signaling—a major inflammatory pathway. Supporting this hypothesis, three NF-κB binding sites have been detected in the *APOBEC3B* promoter, and p65/p50 and p65/c-Rel heterodimers—which are part of the canonical NF-κB pathway—have proved important for *APOBEC3B* transcription [83]. Non-canonical NF-κB signaling may also regulate APOBEC3 expression, as the *APOBEC3B* promoter contains a RelB binding site. Multiple known APOBEC3 inducers such as LPS and IL-4 are also potent NF-κB activators, which further implicates NF-κB as a transcriptional driver of APOBEC3s during inflammation [84].

**Fig. 2:**
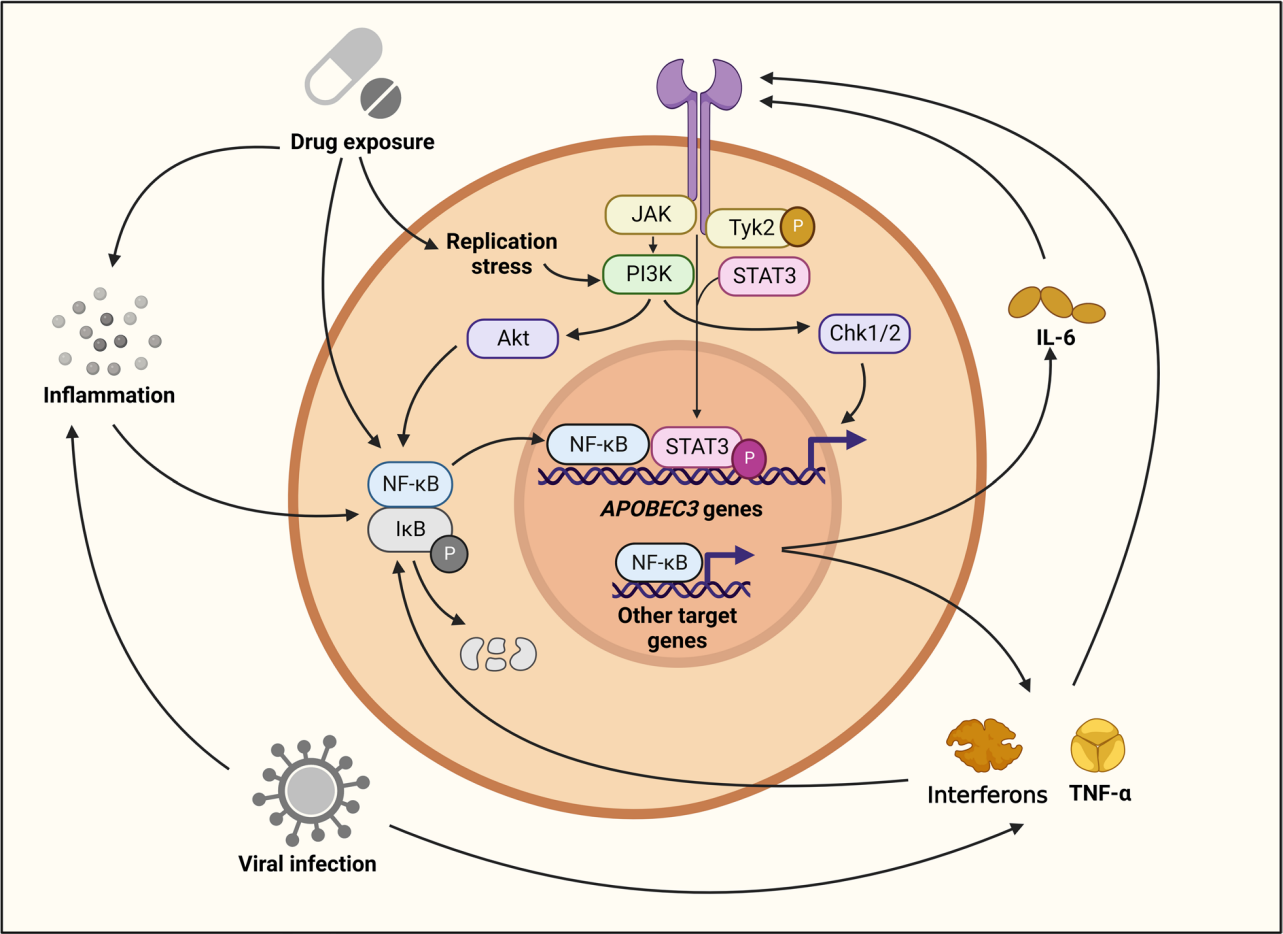
Transcriptional regulation of *APOBEC3* Genes. NF-κB signaling is a shared pathway for endogenous and exogenous APOBEC3 triggers. To induce APOBEC3s, NF-κB signaling acts directly via transcription of *APOBEC3*s and indirectly through the transcription of other inflammatory mediators. Key inflammatory mediators include interferons, TNF-α, and IL-6, which can drive *APOBEC3* transcription through NF-κB and JAK/STAT signaling. Additionally, replication stress can activate NF-κB signaling via PI3K/Akt to promote *APOBEC3* expression

NF-κB signaling may also increase APOBEC3 expression indirectly via the transcription of pro-inflammatory mediators. For example, the NF-κB target gene *IL-6* has been shown to induce *APOBEC3B* expression in hepatocellular carcinoma via JAK/STAT signaling [85, 86]. Similarly, TNF-α has been found to promote *APOBEC3A* expression in keratinocytes [87, 88]. Corroborating these findings, a study of cholangiocarcinoma and gallbladder cancer found upregulation of both APOEBC3A and APOBEC3B with IL-6 and TNF-α exposure [43]. Additionally, IFN-γ has been implicated as a driver of *APOBEC3B* expression in bladder tumors and lung adenocarcinoma [46, 89]. NF-κB signaling—with the ability to act both directly through *APOBEC3* transcription and indirectly through other inflammatory mediators—is thus a likely driver of APOBEC3-mediated mutagenesis in cancer (Fig. 2). This APOBEC3-inducing role of NF-κB may be especially important in immunologically “hot” tumors with highly inflamed microenvironments.

### Drug exposure and replication stress

**Fig. 3:**
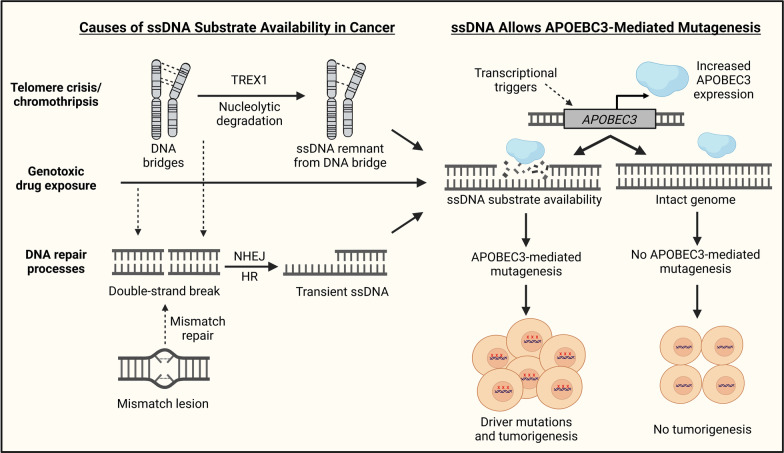
Two-factor model for APOBEC3-mediated mutagenesis. In a two-factor model, APOBEC3-mediated mutagenesis needs both induction of APOBEC3 expression and availability of ssDNA. Factors such as drug exposure, telomere crisis, and DNA repair processes can generate ssDNA, while transcriptional triggers can upregulate APOBEC3 expression [94–96]

Exposure to certain drugs can also trigger APOBEC3-mediated mutagenesis, and chemotherapies such as bleomycin, cisplatin, etoposide, 5-fluorouracil, gemcitabine, hydroxyurea, aphidicolin, and camptothecin have all been shown to induce *APOBEC3* expression in cancer cell lines [1, 61, 90, 91]. NF-κB signaling likely mediates APOBEC3 induction in response to some of these drugs, but replication stress and PI3K signaling may also play major role (Fig. 2) [1, 90, 92, 93]. In addition to increasing APOBEC3 expression, genotoxic drugs may further fuel APOBEC3-mediated mutagenesis by inducing genomic damage and thereby generating ssDNA substrates (Fig. 3).

Even in the absence of genotoxic drugs, cancer cells may experience APOBEC3-inducing replication stress due to accumulated DNA damage and specific oncogenic mutations. In breast cancer, for example, *PTEN* depletion and *HER2* amplification have been shown to induce replication stress and increase APOBEC3B activity in vitro [90]. In lung cancer, the loss of *FHIT1*—a common genetic alteration that causes replication stress—was associated with a higher APOBEC3-induced mutation burden [53]. These replication stress-inducing mutations may be especially strong triggers of APOBEC3-mediated mutagenesis, as they cause persistent cellular changes in contrast with the more transient APOBEC3 induction following viral infection or drug exposure. In a positive feedback loop, increased APOBEC3 expression may exacerbate replication stress due to additional DNA damage, slowed replication forks, and cell cycle arrest [52, 97–99].

In the context of replication stress, APOBEC3s—particularly APOBEC3B—may be especially prone to causing kataegis rather than more diffuse mutation patterns. Accordingly, APOBEC3B has been shown to induce kataegis in cancer cell lines during telomere crisis [94]. APOBEC3B was similarly associated with kataegis in p53-defecient cell lines, and a pan-cancer analysis found that APOBEC3B expression was positively correlated with kataegis [31, 99].

### Somatic and germline alterations

Somatic mutations in multiple genes have been associated with increased APOBEC3-mediated mutagenesis, but whole-genome sequencing analyses have not detected recurrent somatic single-nucleotide variants in the coding or regulatory regions of *APOBEC3*s [100, 101]. Somatic mutations that increase the deamination activity of APOBEC3s are thus unlikely, as are mutations that alter *APOBEC3* expression via increased promoter or enhancer activity. However, increased *APOBEC3* expression may occur due to copy number amplification in cancers. Although only noted in one cancer type, *APOBEC3* copy number variation was found in ~ 30% of lung tumors [36, 102]. This genetic alteration was associated with increased *APOBEC3B* expression and a higher APOBEC-induced mutation burden [36].

Germline variants at the *APOBEC3* gene locus appear to be more common and can influence APOBEC3 expression and cancer risk. For example, the single-nucleotide polymorphism (SNP) rs1014971—which is located upstream of the *APOBEC3* gene cluster—is associated with increased *APOBEC3B* expression, enrichment of APOBEC3-induced mutations, and higher bladder cancer risk [61, 63, 103]. In breast cancer, a deletion polymorphism that generates an *APOBEC3A/B* chimera is associated with increased disease risk, more APOBEC3-induced mutations, and poor tumor differentiation (a sign of negative prognosis) [47, 61, 62, 64, 104, 105]. The same deletion polymorphism may contribute to APOBEC3-induced hypermutation in acute lymphoblastic leukemia [62]. In lung cancer, the combination of six SNPs that define *APOBEC3H* haplotype I have been associated with increased disease risk [65] (Table 2). Genetic variation within this haplotype may further enhance lung cancer risk [106]. Additionally, the variant rs2267401 has been shown to increase gallbladder cancer and hepatocellular carcinoma risk, likely by increased *APOBEC3B* expression that arises through heightened promoter activity and IL-6 response [43, 86].

**Table 2:** Germline variants affecting APOBEC3s and cancer risk

Germline variant	Genomic location	Risk allele or amino acid	Cancer	Molecular impact*	Effect on cancer risk*	References
rs139293	Exon 2 of *APOBEC3H*	T	Lung	Activity-decreasing amino acid change (R18L) and lower expression of *APOBEC3H* and *APOBEC3C*	Decreased	[107]
rs1014971 Linked proxies: rs17000526 rs1004748	Intergenic region ~ 25 kb from *APOBEC3A*	T	Bladder	Increased *APOBEC3B* expression and more APOBEC3-induced mutations	Increased	[61]
				N/A	Increased	[63]
				N/A	Increased overall and for high-risk tumors	[103]
			Breast	Increased *APOBEC3B* expression but no association with APOBEC3-induced mutation burden	Moderately increased	[61]
rs2267401	*APOBEC3B* promoter	G	Bile duct	Increased *APOBEC3B* promoter activity	Increased	[43]
			Liver	Increased *APOBEC3B* expression with stronger promoter response to IL-6 and more APOBEC3-induced HBV mutations	Increased	[86]
			Gallbladder	Decreased *APOBEC3B* promoter activity	Decreased	[43]
rs12157810	*APOBEC3A* promoter	C	Bile duct	Increased *APOBEC3A* promoter activity	Decreased	[43]
			Gallbladder	Increased *APOBEC3A* promoter activity	Decreased	[43]
			Renal	Higher APOBEC3A expression and increased promoter sensitivity to TNF-α	Decreased	[108]
			Colorectal	No change in promoter responsiveness to TNF-α, but decreased response to IL-6	N/A	[108]
*APOBEC3H* Haplotype I	*APOBEC3H*		Lung	Gly105 creates unstable APOBEC3H protein with increased nuclear localization	Increased	[65]
rs34522862	Exon 2	Asn				
rs139293	Exon 2	Arg				
rs139297	Exon 3	Gly				
rs139299	Exon 3	Lys				
re139298	Exon 3	Lys				
rs139302	Exon 4	Glu				
rs139298	*APOBEC3H* Exon 3	Glu	Lung	K121E within APOBEC3H haplotype I destabilizes protein, potentially creating fewer mutations to avoid immune response	Decreased relative to standard haplotype I	[106]
APOBEC3A/B chimera Proxy: rs12628403	Coding region of *APOBEC3B*	~ 30 kb deletion	Breast	Enrichment of APOBEC3-induced mutations	Increased	[61]
				Increased APOBEC3-induced mutation load	N/A	[62]
				Higher abundance of tumor-infiltrating immune cells	Increased	[109]
				N/A	Increased for higher histological grade	[64]
				Hypermutated phenotype with immune activation	N/A	[105]
			Leukemia	Hypermutated tumor genome	N/A	[62]
			Ovarian	N/A	Decreased	[110]
			Bladder	No association	No association	[61]
APOBEC3B indel	Exon 5 of *APOBEC3B*	Deletion	Breast	N/A	Increased	[111]

^*^N/A indicates that the study did not test for molecular impact or clinical association

In contrast, the same SNP rs2267401 was associated with decreased risk of cholangiocarcinoma and lower *APOBEC3B* promoter activity in this tumor type, likely due to overexpression of the transcriptional repressor TFAP2A [43]. Another variant, rs12157810, has also been associated with lower cholangiocarcinoma and gallbladder cancer risk, although it was found to increase *APOBEC3A* promoter activity [43]. Concordant results were observed in renal cancer [108]. Another SNP rs139293 has been associated with decreased risk of lung cancer [107]. Located in an exon, this variant creates a potentially activity-decreasing amino acid change in APOBEC3H and may lower the expression of *APOEBC3H* and *APOBEC3C* [107] (Table 2).

### Retrotransposon activity, telomere crisis, and DNA damage

Cancer cells have generally unstable genomes that can fuel APOBEC3-mediated mutagenesis through multiple distinct pathways. Specifically, genomic instability may promote retrotransposon activity and thereby trigger episodic bursts of APOBEC3-mediated mutagenesis [112]. Genomic instability is also associated with telomere crisis and chromothripsis, which have been shown to generate ssDNA breakpoints deaminated by APOBEC3B [94] (Fig. 3). APOBEC3-mediated mutagenesis can be triggered by upregulation of APOBEC3s during DNA damage-induced replication stress and associated mismatch repair processes such as non-homologous end joining (NHEJ), and homologous recombination (HR) that generate ssDNA intermediates [90, 95, 96, 113–116] (Fig. 3). Since APOBEC3s can deaminate lagging strand templates, the rapidly dividing cancer cells can have frequently exposed ssDNA and thereby fuel APOBEC3-mediated mutagenesis [14].

These endogenous processes in cancer cells may drive the continued acquisition of APOBEC3-induced mutations late in tumor evolution without exposure to exogenous triggers [81]. The importance of DNA damage and ssDNA substrate availability may also explain why APOBEC3s are expressed at low levels in many healthy tissues and do not typically cause considerable tumor genome-like mutation patterns. Significant APOBEC3-induced mutation loads have not been detected in healthy esophageal or endometrial gland tissue, though low levels of such mutations have been found in a subset of non-cancerous intestinal crypts and bronchial epithelial cells [59, 117–120]. A two-factor model for APOBEC3 activity is thus plausible in many cancers, with elevated APOBEC3 expression and pre-existing DNA damage both being required for APOBEC3-mediated mutagenesis (Fig. 3).

### Smoking?

Smoking is a major risk factor for multiple cancers with high APOBEC3-induced mutation loads. For example, smoking accounts for over 50% of bladder cancer risk and is estimated to cause 80–90% of lung tumors [121, 122]. Smoking is most strongly associated with mutational signatures 4, 5, and 29, but multiple studies have tested whether tobacco exposure is also linked with the APOBEC3-induced signatures 2 and 13 [24–26, 123, 124]. However, the results have been contradictory. In an analysis of lung adenocarcinoma, signatures 2 and 13 were enriched in tumors from smokers [123]. In bladder cancer, signature 13 was enriched in tumors from former smokers [125]. However, a separate analysis of only muscle-invasive bladder tumors found that signature 13 was enriched in non-smokers and negatively correlated with signature 5 [124]. Additionally, an in vitro study in normal human urothelium found that that exposure to benzo[a]pyrene—a procarcinogen in cigarette smoke—did not induce signature 2 or13. However, this study has limitations in that it only used one procarcinogen, while tobacco smoke includes approximately 60 carcinogens [126].

If smoking does increase APOBEC3-mediated mutagenesis, the effect is likely due to generalized DNA damage that increases ssDNA substrate availability [123]. Although the nicotine component of tobacco can induce inflammation and NF-kB signaling, there is no evidence that tobacco smoke directly increases APOBEC3 expression. A single study of non-small cell lung cancer tested this hypothesis but found no association between smoking and *APOBEC3B* mRNA expression [54]. However, the prevalence of smoking as a common risk factor for cancers where APOBEC3-mediated mutagenesis is widely operational might make it hard to decern any interactions between smoking and APOBEC3s.

## Impact of APOBEC3s through mutagenic and non-mutagenic pathways

### APOBEC3-induced oncogenic coding mutations

APOBEC3-induced mutations are ubiquitous in cancer and can drive carcinogenesis by activating oncogenes or inactivating tumor suppressors (Table 3). For example, the APOBEC3-induced *FGFR3* S249C mutation—the most common *FGFR3* mutation in bladder cancer—causes constitutive activation of the encoded growth factor receptor to promote cell proliferation [127, 128]. Although less recurrent in other tumor types, S249C has also been detected in lung, cervical, and head and neck cancers [129–132]. In addition to activating receptors, APOBEC3-induced mutations can affect downstream signaling pathways to generate highly oncogenic mutational synergies. The APOBEC3-induced *PIK3CA* E545K mutation, for example, causes aberrant activation of the growth-promoting PI3K pathway and has been detected in bladder, breast, cervical, colorectal, esophageal, head and neck, and lung tumors [39, 132–137]. The highly similar *PIK3CA* E542K mutation has been found in bladder, breast, cervical, colorectal, esophageal, head and neck, and lung cancers [39, 132, 133, 135, 137–140].

**Table 3:** Recurrent APOBEC3-induced somatic mutations and copy number alterations in cancer

Mutation type	Gene name	Gene type	Amino acid alteration	Cancer type(s)	References
Coding	EGFR*	Oncogene	T790M	Lung	[82, 141]
	*FGFR3*	Oncogene	S249C	Bladder, cervical, HNSCC, and lung	[127, 129–132]
	*MEK2*	Oncogene	L46F	Melanoma	[82, 142]
	*PIK3CA*	Oncogene	E545K	Bladder, breast, cervical, colorectal, esophageal, HNSCC, and lung	[39, 132–137]
	*PIK3CA*	Oncogene	E542K	Bladder, breast, cervical, colorectal, esophageal, HNSCC, and lung	[39, 132, 133, 135, 137–140]
	*FBXW7*	Tumor suppressor	R505G	HNSCC, upper digestive tract, urinary tract, and lung	[132, 143]
Non-coding regulatory	*ADGR6/GPR126*	Oncogene	N/A	Bladder	[33, 144, 145]
	*TBC1D12*	Oncogene		Bladder and breast	[144, 147]
	*LMO1*	Oncogene		T-Cell acute lymphoblastic leukemia	[146]
	*PLEKHS1*	Oncogene		Bladder and breast	[144, 147]
	WDR74**	Oncogene		Bladder and breast	[144, 147]
	*LEPROTL1*	Unknown		Bladder and breast	[144, 155]
Copy number amplification	*AGAP2*	Oncogene	N/A	Glioma	[51]
	*CDK4*	Oncogene			
	*EGFR*	Oncogene			
	*MAPKAPK2*	Oncogene			
	*USP15*	Oncogene			
Not specified***	*EGFR*	Oncogene	N/A	Bladder, breast, HNSCC, and lung	[148]
	*PTEN*	Tumor suppressor			
	*TP53*	Tumor suppressor			

^*^C-to-T mutation that is not in the preferred APOBEC3 motif but has been hypothesized to be a rare APOBEC3-induced mutation that is highly selected for given its strong oncogenic effects

^**^Mutation has a less clear association with APOBEC3s and may arise from other mutational processes

^***^Subclonal mutations in cancer driver genes, but the mutations are not specified as coding or non-coding

APOBEC3-induced and other oncogene-activating mutations may allow cancer cells to proliferate unchecked in the presence of additional mutations in tumor suppressor genes—some of which arise from APOBEC3-mediated mutagenesis. For example, the inactivating R505G *FBXW7* mutation is attributable to APOBEC3s and has been detected in HNSCC, upper digestive tract cancers, urinary tract cancers, and lung cancers [132, 143]. Subclonal APOBEC3-induced mutations have also been observed in the tumor suppressor genes *PTEN* and *TP53* in bladder, breast, head and neck, and lung tumors [148].

Other mutational processes can also inactivate tumor suppressors, and high APOBEC3 activity may generate a selective pressure favoring such mutations. APOBEC3s create a high overall tumor mutation burden, so cells with impaired DNA damage response (DDR) due to tumor suppressor mutations are more likely to avert apoptosis and continue proliferating. Accordingly, *TP53* mutations—which are mostly non-APOBEC3-induced—were more common  in bladder cancers, lung adenocarcinomas, and B-cell lymphoma cell lines with high burden of APOBEC3-induced mutations [46, 89, 149]. Similarly, high APOBEC3B expression has been associated with more p53 mutations in breast cancer and adrenocortical carcinoma [22, 44]. In addition to selection pressure, this trend could arise from higher APOBEC3 expression in p53-mutated tumors since p53 may suppress *APOBEC3B* transcription via p21 and DREAM proteins [150].

### Recurrent APOBEC3-induced non-coding mutations

Some APOBEC3-induced mutations in non-coding regulatory regions are also highly recurrent and may contribute to tumor development by modulating the expression of cancer-related genes (Table 3). For example, many bladder and breast tumors harbor APOBEC3-induced “twin mutation hotspots” in the promoters of *PLEKHS1* and *TBC1D12*, which are potential oncogenes associated with invasive disease and poor prognosis [33, 144, 147, 151, 152]. APOBEC3-induced *ADGR6/GPR126* enhancer mutations implicated in angiogenesis have also been detected in bladder cancer [33, 145]. Other genes with non-coding mutations attributable to APOBEC3s in bladder cancer include *LEPROTL1* and potentially the tumor suppressor *WDR74* [144, 153, 154]. Additionally, many T-cell acute lymphoblastic leukemias harbor an APOBEC3-induced mutation upstream of the transcription start site for the oncogene *LMO1* [146]. Several such mutations were found functional and having potential oncogenic role in breast cancer [155]*.* However, what triggers APOBEC3-mediated mutagenesis in non-coding intergenic and promoter regions has not been decerned, but it likely occurs during DNA replication, and transcription initiation.

In addition to generating point mutations in regulatory regions, APOBEC3s may further dysregulate gene expression by promoting increased oncogene copy number (Table 3). For example, higher APOBEC3 expression has been associated with increased copy number variation of multiple Ras/MAPK regulatory genes such as *ATP2B4*, *MAPKAPK2*, and *USP15* in glioma [51]. Amplification of *EGFR* and *CDK4*—both known oncogenes—has also been observed in APOBEC3-high gliomas [51]. The mechanisms through which APOBEC3s facilitate copy number changes remain unknown. Though it is plausible that APOEBC3-induced kataegis promotes chromosomal instability and double strand breaks, creating opportunities for copy number changes [156, 157].

### Tumor evolution

Although some APOBEC3-induced mutations are likely important for initial tumor formation, many occur later in tumor evolution. Supporting this paradigm, episodic bursts of APOBEC3-mediated mutagenesis were observed during prolonged culture of numerous cancer cell lines [81, 112]. This repeated APOBEC3 activity can create a high overall tumor mutation burden and fuel subclone heterogeneity in a tumor context. Accordingly, APOBEC3s have been identified as primary drivers of subclonal mutations in bladder, breast, head and neck, and lung cancers [48, 148]. In bladder cancer, over 45% of subclonal mutations in driver genes may be attributable to APOBEC3s [148]. Additionally, APOBEC3-induced mutation load has been strongly associated with tumor heterogeneity in metastatic thoracic cancers [89]. APOBEC3-induced tumor heterogeneity can promote resistance to cancer therapies. While the continued acquisition of signatures 2 and 13 is often part of natural tumor evolution, chemotherapy treatment may further fuel APOBEC3-mediated mutagenesis by triggering APOBEC3 expression and inducing DNA damage [1, 81, 90, 91].

Specific therapy resistance mutations can also arise due to APOBEC3 activity. For example, the APOEBC-induced *MEK2* L46F mutation may confer resistance to BRAF inhibitors such as vemurafenib and dabrafenib in melanoma [82, 142]. In lung cancer, the potentially APOBEC3-induced C > T *EGFR* T790 mutation can promote resistance to the EGFR inhibitors gefitinib and erlotinib [82, 141]. Similarly, some APOEBC3-induced mutations observed in relapsed refractory multiple myeloma may contribute to acquire therapy resistance [158].

### Non-mutagenic pathways

APOBEC3-induced mutations are significant drivers of tumorigenesis, but APOBEC3s can also play role in cancer through non-mutagenic pathways. Exemplifying the importance of these pathways, a study of hepatocellular carcinoma found that overexpression of catalytically inactive APOBEC3B increased cell proliferation, cell migration, and cell invasion in vitro [159]. In the presence of K-Ras mutations, APOEBC3A may also promote STING-dependent metastasis and chromosomal instability based on a mouse model of pancreatic ductal adenocarcinoma [157].

Overexpression of catalytically inactive APOBEC3B has also been associated with more frequent “G1 escape,” suggesting that APOBEC3B contributes to cell cycle dysregulation [159]. Similar APOBEC3B-mediated cell cycle progression has been observed in bladder cancer [159, 160]. APOBEC3s can also inhibit cell death via multiple mechanisms: APOBEC3G was shown to inhibit anoikis via Akt activation in pancreatic cancer, and APOBEC3B may decrease cell death in gastric cancer by inhibiting PDCD2 function and lowering ATM and Chk1/2 activity [38, 49].

Additionally, APOBEC3s may affect the expression of oncogenes and tumor suppressors through epigenetic-coupled mechanisms. For example, APOBEC3B has been shown to drive estrogen receptor (ER) overexpression in breast cancer through transient chromatin remodeling [161]. Providing further evidence of APOBEC3-mediated epigenetic regulation, high APOBEC3B expression has been associated with greater LINE1 methylation (a proxy for global DNA methylation) in esophageal cancer [39].

### Modulation of the tumor immune microenvironment

APOBEC3s can also impact tumor growth through non-mutagenic pathways that shape the tumor immune microenvironment (Table 4). APOBEC3s are immunosuppressive in some cancers, and higher *APOBEC3B* expression has been associated with less immune cell infiltration in adrenocortical carcinoma and gastric cancer [37, 162]. APOBEC3 expression has also been associated with greater infiltration of immunosuppressive mediators such as myeloid-derived suppressor cells (MDSCs) and tumor-associated macrophages (TAMs) in a mouse model of hepatocellular carcinoma [163].

**Table 4:** Immune-modulating effects of APOBEC3s by cancer type

Cancer type	APOBEC3(s)*	Effect on immune system	Immune features associated with APOBEC3s	Study type	References
Adrenal	3B	Suppressive	Decreased immune cell infiltration	Patient	[162]
Gastric	3B	Suppressive	Fewer CD8+ T cells expressing effector antigens and immune checkpoint proteins	Patient	[37]
Liver	3B	Suppressive	More immunosuppressive mediators such as MDSCs and TAMs	Mouse	[163]
Breast	3B	Activating	More CD4+ and CD8+ T cells with increased IFN-γ and cytotoxic granzyme B production. Fewer T-reg, Th2, and TAM cells	Mouse	[164]
	3B	Activating	More tumor-infiltrating lymphocytes	Patient	[165]
	3B	Activating	Immune activation in pan-cancer analysis	Patient	[162]
	3C-H	Activating	More CD8+ T-cells with greater receptor diversity and cytolytic activity	Patient	[48]
Bladder	3B	Activating	Enrichment of immune-related signatures, including IFN-γ	Patient	[46]
	Mutation burden	Activating	Higher neoantigen loads, activated CD4+ T cells, CD8+ T cells, and M1 cells. Fewer MDSCs and naïve CD4+ T cells	Patient	[160]
	3B	Activating	More cytotoxic T-cells with a higher CD8+/CD3+ ratio	Patient	[35]
Glioma	3B, 3C, 3D, 3F, 3G, 3H	Activating	Increased myeloid cell infiltration, IFN-γ signaling, and PD-L1/2 expression	Patient	[51]
Lung	3B	Activating	More activated CD4+ T cells, CD8+ T cells, and NK cells. Fewer regulatory T cells	Patient	[166]
	3B	Activating	Upregulation of immune genes and more T cell infiltration to the tumor site	Patient	[36]
	Mutation burden	Activating	Enrichment of interferon pathways	Patient	[89]
Melanoma	3B	Activating	Immune activation in a multi-cancer analysis	Patient	[162]
Ovarian	3B	Activating	Greater tumor immune cell infiltration	Patient	[41]
	3G	Activating	Higher immune cell counts in the tumor microenvironment	Patient	[167]

^*^Where applicable, “mutation burden” refers to APOBEC3-induced mutation burden

The opposite effect has been observed in other cancer types, with APOBEC3s promoting immune activation. In a pan-cancer analysis, high APOBEC3B expression was associated with increased immune activation in cutaneous melanoma and breast cancer [162]. Additional studies in breast cancer have found similar results. High *APOBEC3B* expression has been associated with more tumor infiltrating lymphocytes, and APOBEC3B induction drove a robust T cell-mediated immune response in a mouse model [164, 165]. APOBEC3C-H levels were also correlated with more CD8+ T cells in the tumor microenvironment, increased T cell receptor diversity, and greater cytolytic activity [48, 64].

Similar immune activation has been observed in bladder cancer, with multiple studies detecting increased immune signatures and interferon signaling in APOBEC3-high tumors [35, 46, 160]. In lung cancer, T cell-mediated immune activation was linked with high *APOBEC3B* expression or high APOBEC3-induced mutation loads [36, 166]. In ovarian cancer, elevated *APOBEC3B* and *APOBEC3G* expression have been associated with greater immune cell infiltration [41, 167]. Although not in the tumor itself, increased *APOBEC3A* expression has been shown to shift macrophage polarization to a pro-inflammatory, immune-activating state [168].

## Clinical and therapeutic significance of APOBEC3s in cancers

### Association with poor prognosis in multiple cancers

Immunosuppression may synergize with APOBEC3-mediated mutagenesis in some cancers, allowing oncogenic mutations to accumulate and drive tumorigenesis while averting the host immune response. In accordance with this model, studies in adrenocortical carcinoma and gastric cancer—which both show immunosuppression with APOBEC3s—have found that higher *APOBEC3B* expression is correlated with shorter survival (Tables 4 and 5) [37, 38, 44]. Elevated APOBEC3 expression has also been associated with adverse clinical outcomes in nasopharyngeal carcinoma, clear cell renal carcinoma, and neuroendocrine tumors, though little is known about APOBEC3-mediated immune effects in these cancers (Table 5) [55, 169].

**Table 5:** Association of APOBEC3 expression and APOBEC3-induced mutations with clinical outcomes by cancer type

Cancer type	APOBEC3(s)*	Outcome type	Immunotherapy context?	Clinical outcome	References
Adrenal	3B	Adverse	No	Shorter overall and disease-free survival	[44]
Bladder	3B	Positive	No	Longer survival with APOBEC3B-enhancing SNP (rs101497)	[61]
	Mutation burden	Positive	No	Longer survival in muscle-invasive disease cohort	[170]
	Mutation burden	Positive	No	Longer survival	[46]
	3D and 3H	Positive	No	Better overall survival	[171]
	3B	Adverse	No	Shorter progression-free survival with mutagenic A3B1 isoform	[1]
	Mutation burden	Adverse	No	Higher risk of class 2a tumors and poor outcomes for non-muscle invasive disease	[172]
	3A and 3B	Adverse	No	Increased risk of class 2a tumors	[173]
	Mutation burden	Positive	Yes	Longer survival overall and better response to immune checkpoint blockade therapy	[160]
	Mutation burden	Positive	Yes	Increased responsiveness to immunotherapy	[174]
Breast	3A	Adverse	No	Advanced tumor stage, higher histological grade, and more lymph node involvement	[64]
	3A	Adverse	No	Shorter overall survival	[175]
	3B	Adverse	No	Higher tumor grade with lymph node involvement and worse prognosis	[105]
	3B	Adverse	No	Higher nuclear grade and more frequent lymph node metastasis	[176]
	3B	Adverse	No	Shorter disease-free, metastasis-free, and overall survival. Lower overall survival in ER+ subgroup	[177]
	3B	Adverse	No	Shorter survival in ER+ but not ER- group	[161]
	3B	Adverse	No	Lower recurrence-free survival in a combined analysis and ER+ subgroup	[178]
	3A/B	Adverse	No	Higher histological grade with 3A/B chimera	[64]
	3A-H	Mixed	No	Aggressive pathology and shorter survival (3A and 3B). Improved survival (3C-H)	[48]
	3B	Mixed	No	Shorter replace-free, metastasis-free, and overall survival (mRNA). Increased disease-free survival (protein)	[165]
	3B	Mixed	No	Higher nuclear grade and Ki-67 labeling index, but improved responsiveness to neoadjuvant chemotherapy	[179]
	Mutation burden	Adverse (overall)	Yes	Shorter survival with first-line endocrine and CDK4/6i therapy, but improved response to immune checkpoint blockade therapy	[180]
		Positive (immunotherapy)			
	Mutation burden	Positive	Yes	Improved response to immunotherapy	[181]
Esophageal	3B	Adverse/none	No	Higher histological grade	[39]
Gastric	3B	Adverse (overall)	No	Shorter overall and progression-free survival, but improved response to fluorouracil-based adjuvant chemotherapy and PD-1 blockade therapy	[37]
		Positive (immunotherapy)	Yes		
	3B	Adverse	No	Aggressive pathology and poor survival	[38]
Glioma	3B, 3C, 3D, 3F, 3G, 3H	Adverse	No	Poor prognosis, especially in the low-grade subtype	[51]
Head and neck	Mutation burden	Positive	Yes	Improved responsiveness to immunotherapy	[174]
Lung	3B	Adverse	No	Shorter survival	[182]
	3B	Adverse	No	Shorter disease-free and overall survival with more with lymph node involvement and higher TNM stage	[183]
	3B	Adverse	No	Shorter overall survival	[175]
	Mutation burden	Adverse	No	Worse overall survival	[184]
	Mutation burden	Adverse	No	Signature 2 (but not 13) burden associated with poor prognosis	[185]
	3B	None	No	No association with pathological features or clinical outcomes	[54]
	Mutation burden	Positive	Yes	Increased response to immune checkpoint blockade therapy	[166]
	3B	Positive	Yes	Improved responsiveness to immune checkpoint blockade therapy	[36]
Nasopharyngeal	3B	Adverse	No	Aggressive disease and poor outcomes	[55]
Neuroendocrine tumor	3B	Adverse	No	More lymph node metastasis	[40]
Ovarian	Mutation burden	Positive	No	Improved progression-free and overall survival	[186]
	3B	Adverse	No	Shorter disease-free and overall survival	[42]
	3B	Positive	No	Longer progression-free (significant) and overall (not significant)	[41]
	3G	Positive	No	Favorable clinical outcomes with A3G, but no association for A3B	[167]
Pancreatic	3G	None	No	No association with tumor stage, tumor type, or prognosis	[49]
Renal	3B	Adverse	No	More frequent recurrence	[169]

^*^Where applicable,** “**mutation burden” refers to APOBEC3-induced mutation burden

High APOBEC3 expression or induced mutation burden may also predict adverse outcomes in breast cancer, although some reports have found inconsistent results (Table 5). Since APOBEC3s induce immune activation in breast cancer, additional factors must overcome this heightened immune response to drive tumorigenesis (. 4). Greater estrogen receptor activation (ER) is a likely mechanism since APOBEC3B has been shown to promote ER overexpression in breast cancer [161]. Accordingly, associations between high APOBEC3B expression and adverse clinical outcomes are stronger in ER+ disease (Table 5).

**Fig. 4:**
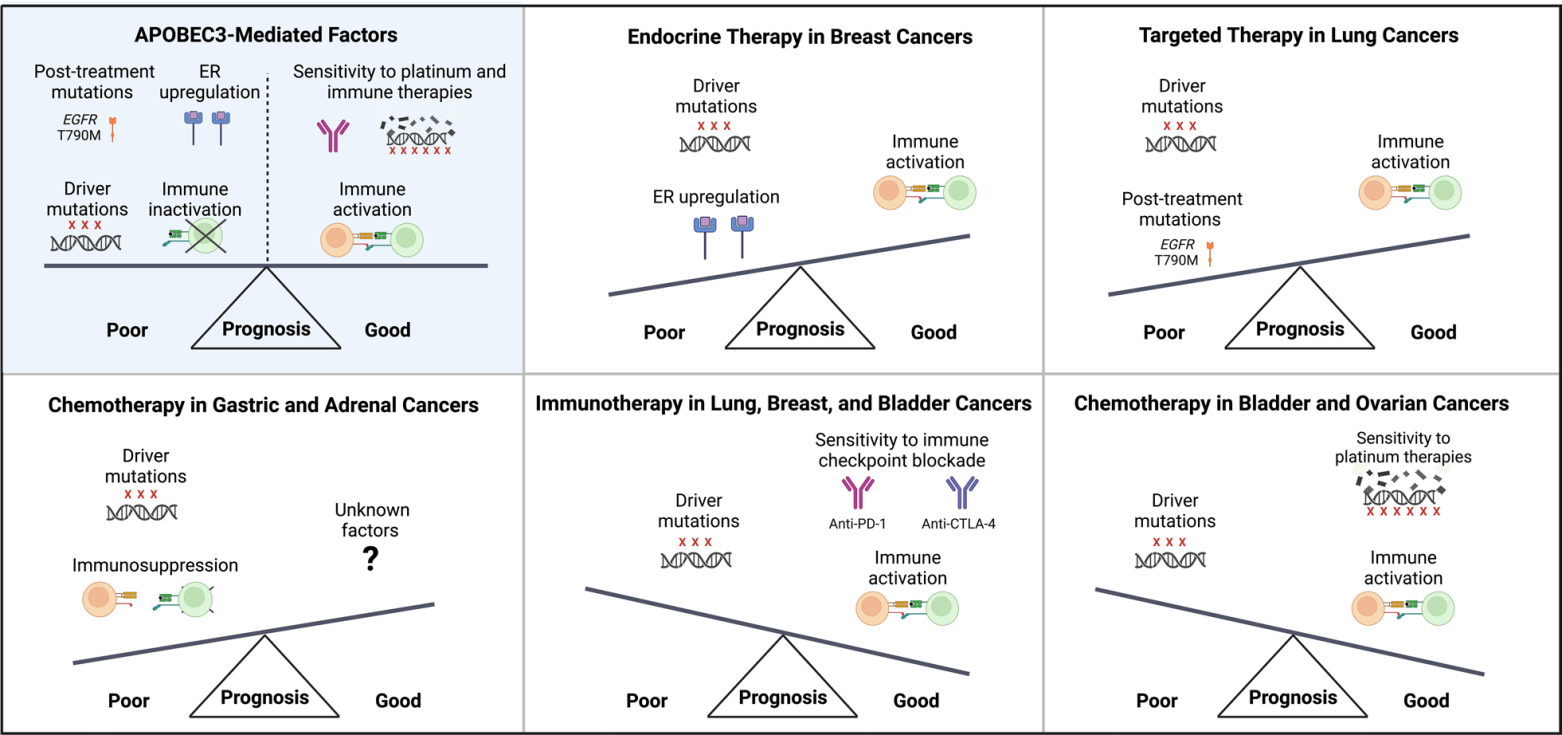
Effects of APOBEC3-induced factors on prognosis varies  among cancer types. APOBEC3s can affect tumorigenesis through mutagenic and non-mutagenic pathways, which may have opposing effects on disease course. The relative strength of these effects may determine the prognostic significance of APOBEC3s, explaining the varied clinical impact of APOBEC3s by cancer type (Table 5). Examples for specific cancer types are shown.

APOBEC3s have similarly deleterious effects in traditionally treated lung cancers. Outside of the immunotherapy context, multiple studies have found that *APOBEC3B* expression or induced mutation burden is associated with aggressive disease or poor prognosis (Table 5). In lung cancer, additional factors such as APOBEC3-induced mutations that promote therapy resistance are thus likely to outweigh APOBEC3-mediated immune activation to drive poor clinical outcomes [141] (Fig. 4).

### Association with favorable immunotherapy and platinum-based treatment outcomes

In lung cancer, APOBEC3-mediated immune activation has been associated with improved responsiveness to immune checkpoint blockade therapies (Table 5). Similar results have been observed for immunotherapy response in breast, bladder, and lung cancers, which have all shown immune activation with APOBEC3s (Tables 4 and 5). In these cancers, APOBEC3-mediated immune activation likely synergizes with a high APOBEC3-induced neoantigen load to drive immunotherapy responsiveness (Fig. 4) [164, 187]. APOBEC3s are also associated with improved response to immunotherapy in gastric cancer, but this cancer type has paradoxically shown immunosuppression with APOBEC3s [37].

Although current data are somewhat unclear, APOBEC3s may also facilitate improved bladder cancer survival even with traditional chemotherapy (Table 5). Bladder cancer is often treated with the DNA alkylating agent cisplatin, which may have enhanced activity in the presence of APOBEC3s. Mechanistically, APOBEC3s may mediate cisplatin response by deaminating drug-induced extrahelical cytosines, and APOBEC3B may induce further genotoxic effects during chemotherapy-induced mismatch repair [188]. Additionally, APOBEC3s may generate a high background mutation load that sensitizes cells to additional cisplatin-induced DNA damage. However, the clinical benefit of APOBEC3s in bladder cancer could be limited to later-stage disease since higher APOBEC3B expression may contribute to the progression from non-muscle-invasive to muscle-invasive disease [1].

APOBEC3s have been similarly associated with improved clinical outcomes in ovarian cancers, which are treated like bladder cancer using the platinum-based therapies cisplatin and carboplatin (Table 5). For example, a high APOBEC3-induced mutation load was associated with improved progression-free and overall survival in clear cell ovarian carcinoma patients [186]. High APOBEC3 expression correlated with improved survival in high-grade serous ovarian tumor subtypes [41, 42, 167]. Like in bladder cancer, these APOBEC3-associated survival benefits in ovarian cancer likely arise from immune activation and increased responsiveness to platinum-based therapies (Fig. 4).

### APOBEC3s as biomarkers

APOBEC3 expression and APOBEC3-induced mutation burden have been associated with clinical outcomes in multiple cancers and treatment contexts, which rationalizes their use as prognostic biomarkers (Table 5). Several studies of cisplatin/carboplatin-treated cancers have reported associations between APOBEC3s and favorable outcomes, so APOBEC3s merit further evaluation as biomarkers for this common chemotherapy [41, 46, 61, 167, 170, 186]. APOBEC3s could also be used as biomarkers for immunotherapy given existing data in lung, bladder, and breast cancers [37, 160, 166, 174, 180, 181]. In addition to these clinical associations, multiple pre-clinical studies have bolstered the case for APOBEC3s as potential platinum-based therapy and immunotherapy biomarkers (Table 6).

**Table 6:** Biomarker and therapeutic potential of APOBEC3s

	Treatment or tumor context	Emerging evidence	APOBEC3 metric*	References
*Biomarker*: Predict strong responsiveness *Therapeutic strategy*: Activation of APOBEC3s to sensitize tumor cells to treatment	Immunotherapy	Better response to immune checkpoint blockade therapy in bladder, HNSCC, breast, and lung cancer	Mutation burden and expression	[37, 160, 166, 174, 180, 181]
		Increased PD-L1 expression	Expression (3B)	[36]
		Higher PD-L1 and PD-L2 levels in glioma	Expression	[51]
		Slightly higher sPD-L levels in breast cancer	Expression (3A)	[64]
		A3A, A3D, and A3H correlated with PD-L1 on tumor-infiltrating immune cells, and A3F associated with more PD-L1 on tumor cells	Expression (3A, 3D, 3H, and 3F)	[171]
		APOBEC3 overexpression and APOBEC3-induced kataegis positively correlated with PD-L1 expression	In vitro overexpression	[189]
		Correlation with PD-L1 expression due to induction of PD-L1 via JNK/c-JUN signaling	In vitro overexpression, mutation burden and expression (3A)	[190]
		Increased immune checkpoint blockade responsiveness in a melanoma model	In vivo overexpression (3B)	[187]
		Improved response to anti-CTLA-4 therapy in a HER2-driven breast cancer model	In vivo overexpression	[164]
	Platinum-based chemotherapy	Better survival in bladder cancer, which is often treated with cisplatin**	Mutation burden and expression	[46, 61, 170]
		Improved outcomes in ovarian cancer, which is typically treated with cisplatin or carboplatin**	Mutation burden and expression	[41, 167, 186]
		Increased response to cisplatin in breast cancer cells	In vitro overexpression	[188]
	DDR inhibitors	Increased responsiveness to ATR and Chk1/2 inhibitors in leukemia cells	In vitro overexpression (3A)	[52]
		Higher sensitivity to ATR and Chk1/2 inhibition	In vitro overexpression	[191]
		Greater sensitivity to CHEK1/2 PARP, and WEE1 inhibition in p53-defecient T cells	In vitro overexpression	[99]
		Enhanced sensitivity to PARP inhibitors in pancreatic cancer cells	In vitro overexpression (3A)	[157]
	Tumors with DNA repair deficits	Induction of apoptosis, likely due to high levels of DNA damage	In vitro overexpression (3A)	[43]
*Biomarker*: Predict resistance *Therapeutic strategy*: Inhibition of APOBEC3s to promote therapeutic response or delay resistance	Rapidly evolving tumors	APOBEC3s identified as drivers of tumor heterogeneity and subclonal evolution	N/A	[82]
		APOBEC3-induced subclonal driver mutations in pan-cancer analysis	N/A	[148]
	EGFR inhibitors	APOBEC3s may induce the T790M mutation that confers resistance to EGFR inhibitors in lung cancer	N/A	[82, 141]
	Raf inhibitors	Poor response to Raf inhibitors in glioma	Expression	[51]
		APOBEC3-induced mutations may decrease response to Raf inhibitors in multiple myeloma	N/A	[158]
		APOBEC3-induced *MKE2* L46F promotes resistance to BRAF inhibitors in melanoma	N/A	[82, 142]
	Akt inhibitors	Increased Akt-mediated anoikis inhibition	In vitro overexpression	[49]
	Endocrine therapies	Accelerated resistance to tamoxifen in breast cancer cells through a catalytic mechanism	In vitro overexpression	[192]
	Oncolytic virotherapy	Escape from vesicular stomatitis virus therapy in melanoma models	In vitro and in vivo overexpression	[193]
		Decreased efficacy of oncolytic virus therapy	In vitro and in vivo overexpression	[194]
		Recurrent APOBEC3-induced mutations un resistant cells	In vitro and in vivo overexpression	[187]

*Where applicable, “mutation burden” refers to APOBEC3-induced mutation burden

**Some studies have reported mixed results (see Table 5)

Moreover, APOBEC3s could be particularly fruitful biomarkers for targeted therapies (Table 6). High APOBEC3B expression has been associated with poor response to Raf inhibitors in glioma, and certain APOBEC3-induced mutations may predict resistance to Raf inhibitors and EGFR inhibitors in multiple myeloma and lung cancer, respectively [82, 158]. In ER^+ ^breast cancer, high *APOBEC3B* expression can predict resistance to the endocrine therapy tamoxifen [192]. Based on pre-clinical studies, APOBEC3s may also predict a favorable response to targeted replication checkpoint and DDR inhibitors, likely due to synthetic vulnerabilities that induce cytotoxic DNA damage [52, 99, 157, 191].

Overall, there is compelling evidence that testing patient tumors for APOBEC3 expression or induced mutation burden could guide therapy decisions. However, there is a significant gap from laboratory to clinic, and it will be essential to establish meaningful parameters that define “APOBEC-positivity.” To this end, future studies will need to evaluate APOEBC3 expression or mutational signature scores that can eventually be used in clinical trials. The scoring and profiling systems for PD-L1/PD1 and overall tumor mutation burden can be used for guidance [195–199].

### Inhibition of APOBEC3s for cancer therapeutics

Since APOBEC3s can drive tumorigenesis through mutagenic and not mutagenic pathways, it could be beneficial to inhibit APOBEC3s in specific but likely numerous oncogenic contexts (Table 6). For example, APOBEC3 inhibitors could potentially prevent non-muscle invasive bladder cancer from progressing to muscle-invasive disease, which has a higher APOBEC3-induced mutation burden [1, 200]. APOBEC3 inhibitors could also slow STING-dependent metastasis, especially in combination with emerging STING inhibitors [157, 201]. Inhibiting APOBEC3G could also support anoikis since APOBEC3s have been shown to disrupt this form of cell death [49]. Additionally, APOBEC3 inhibitors could be used especially in cancers where APOBEC3s predict poor prognosis, to limit tumor evolution, subclone heterogeneity, and chemotherapy resistance [82, 148].

APOBEC3 inhibitors are not currently available for clinical use, but recent advancements in resolving the molecular structures of APOBEC3 enzymes have laid important groundwork for the eventual development of this drug class [202]. Multiple compounds with catechol moieties have been shown to inhibit APOBEC3G, with chemical modifications supporting more limited targeting of APOBEC3A [203]. A recently identified small molecule inhibitor that can target catalytic pockets of AID, APOBEC3A and APOBEC3B has also been reported and may guide the design of inhibitors specific to each APOBEC enzyme [205]. In addition to small molecule inhibitors, other potential strategies to reduce APOBEC3 activity include gene-silencing therapies, ssDNA-containing 2′-deoxyzebularine analogues, and alternative splicing modulators [204]. The latter approach is possible because some APOBEC3 isoforms are non-mutagenic, and SF3B1 inhibition has been shown to shift expression to a non-mutagenic *APOBEC3B* isoform by inducing exon 5 skipping [1]. Additionally, alleviation of replication stress via nucleoside supplementation and chk1 inhibition has been shown to reduce APOBEC3B expression in vitro [90].

### Activation of APOBEC3s for cancer therapeutics

**Fig. 5:**
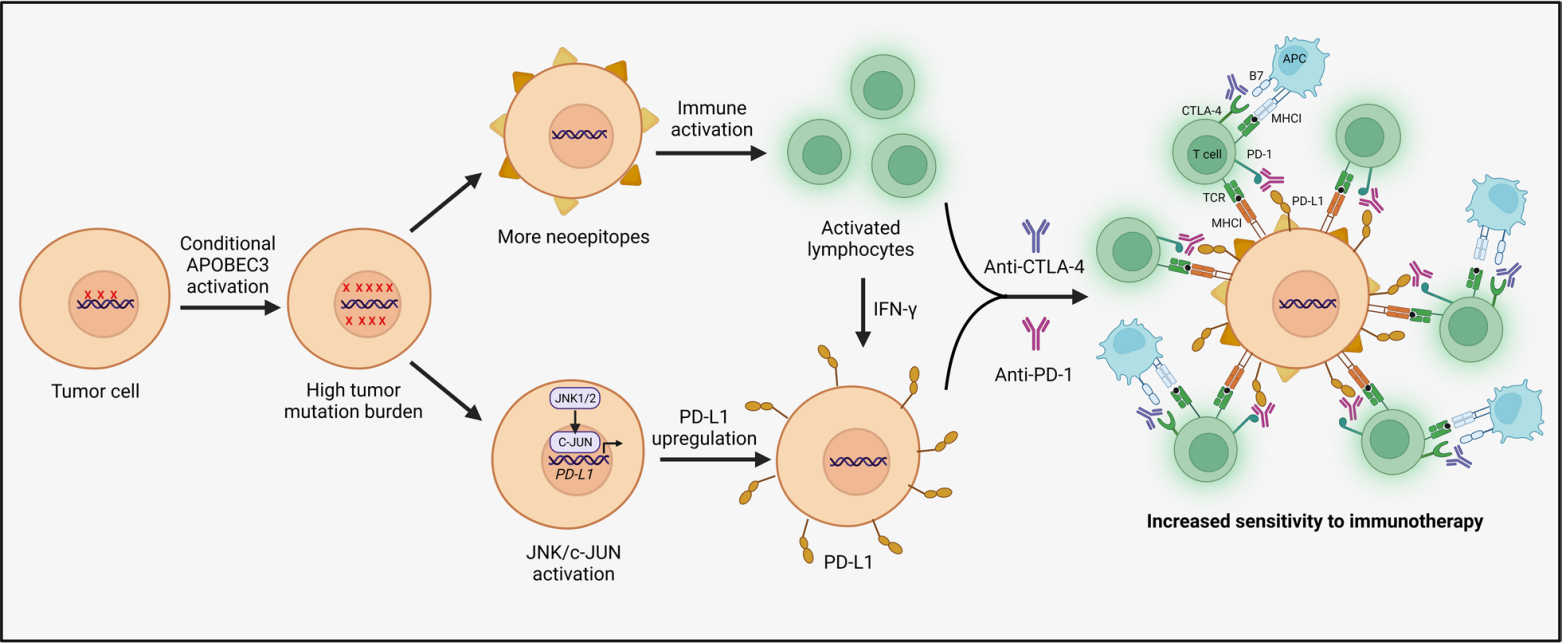
Activation of APOBEC3s to enhance response to immunotherapy. Increasing APOBEC3 activity could increase immunotherapy response through neoepitope formation, increased PD-L1 expression, and immune activation as has been demonstrated by studies using mouse models

While inhibiting APOBEC3s may be beneficial in many cancers, the seemingly counterintuitive strategy of increasing APOBEC3 activity in certain contexts might offer a unique therapeutic opportunity. For example, targeted overexpression of APOEBC3s may be advantageous with immunotherapy agents. Supporting this approach, *APOBEC3B* induction has been shown to increase responsiveness to immune checkpoint blockade therapy in mouse models of melanoma and breast cancer [164, 187]. This APOBEC3-induced sensitization may occur through multiple synergistic pathways (Fig. 5). First, APOBEC3s can generate a high overall tumor mutation burden, leading to neoepitope formation and immune activation in some tumor types [187]. Second, APOBEC3-mediated increases in PD-L1 expression can also enhance immunotherapy response [64, 171, 189]. Mechanistically, APOBEC3s may promote PD-L1 expression through DNA damage that activates JNK/c-JUN signaling [190]. Synergies between APOBEC3s and IFN-γ may further increase PD-L1 expression on cancer cells and additively enhance sensitization to immunotherapy [206, 207].

Since APOBEC3s induce a high level of DNA damage, their targeted overexpression in cancer cells may also be cytotoxic [208]. Activation of APOBEC3A may specifically cause apoptosis in vitro, as it has the highest deamination activity among APOBEC3s [43, 108]*.* Moreover, targeted overexpression of APOBEC3s may be especially cytotoxic to tumors with impaired uracil glycosylase function or loss of the abasic site sensor HCES—mutations that would create synthetic vulnerability [209, 210].

APOBEC3-activating therapies could also effectuate the use of genotoxic drugs. For example, APOBEC3 inducers could prime tumors to respond to platinum-based chemotherapies and DDR inhibitors. As a proof of concept, an in vitro breast cancer study found that inducing *APOBEC3s* in cell lines with low baseline expression significantly increased responsiveness to cisplatin [188]. Overexpression of APOBEC3s was also shown to increase responsiveness to targeted ATR and Chk1/2 inhibitors in acute myeloid leukemia and osteosarcoma cell lines [52, 191]. Similarly, APOBEC3B overexpression sensitized p53-deficient cells to CHEK1/2, WEE1, and PARP inhibition [99]. Increased sensitivity to PARP inhibitors was also observed with APOBEC3A upregulation in pancreatic cancer cells [157].

Despite having therapeutic potential in some contexts, APOBEC3 induction may not facilitate immunotherapy response in all cancers, especially in tumors where higher APOBEC3 expression has been associated with immunosuppression (Table 4). APOBEC3-enhancing drugs must also be explored with caution given the risks of new driver mutations, increased tumor heterogeneity, and chemotherapy resistance. Nonetheless, activation and inhibition of APOBEC3 activity are both promising approaches that could create new treatment options for tomorrow’s clinical landscape.

## Conclusions

APOBEC3s are major mutagenic drivers in cancer, and APOBEC3A and APOBEC3B are likely the most significant mutators among the APOBEC3 superfamily. APOBEC3A and APOBEC3B are overexpressed in many cancers, and their expression correlates with a higher APOBEC3-induced mutation burden. However, the detection of residual deamination activity after knocking out both family members suggests that other APOBEC3s are also active mutators. An improved understanding of how each APOBEC3 contributes to cancer will further elucidate tumor etiology and may become therapeutically relevant for targeting APOBEC3s.

**Fig. 6:**
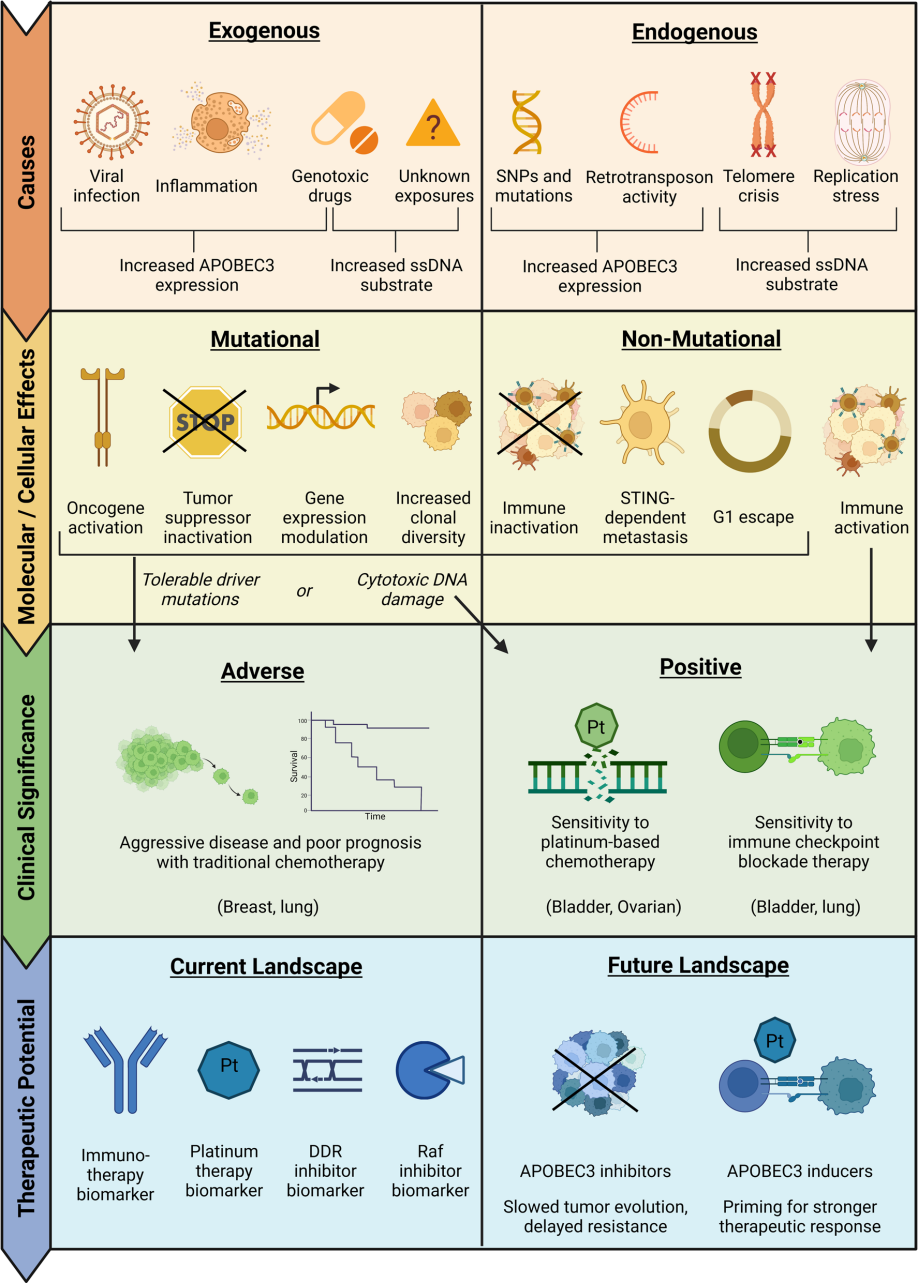
Causes, consequences, and clinical aspects of APOBEC3-mediated mutagenesis in cancer. A variety of exogenous and endogenous factors can induce APOBEC3s, which can then impact cancer growth through mutagenic and non-mutagenic pathways. These APOBEC3-mediated effects may shape clinical outcomes and be relevant in both the current and future therapeutic landscapes.

A more comprehensive understanding of the factors that induce APOBEC3-mediated mutagenesis could also lead to improved cancer prevention strategies. Currently known triggers of APOBEC3 expression include viral infection, inflammation, and certain genotoxic drugs, although increased expression alone may not cause high levels of mutagenesis (Fig. 6). In a two-factor model, ssDNA—which can arise from genotoxic drug exposure, genomic instability, etc.—creates prime mutational substrates for APOBEC3s and facilitates mutagenesis.

Although mutagenesis is central to the role of APOBEC3s in cancer, APOBEC3s can also contribute to tumorigenesis through non-mutagenic pathways such as cell cycle modulation, epigenetic regulation, STING-dependent metastasis, and cell death inhibition (Fig. 6). Additionally, APOBEC3s can modulate the tumor immune microenvironment, with the effect varying by cancer type. APOBEC3s have been associated with immune-activation in breast, lung, and bladder cancer, while APOBEC3-mediated immunosuppression has been observed in liver cancer, gastric cancer, and adrenocortical carcinoma.

The immune-modulating effects of APOBEC3s likely impact disease course, but APOBEC3-mediated immune-activation does not always translate to improved clinical outcomes since other factors such as driver mutations and cell death inhibition can have opposing effects. APOBEC3-mediated immune activation, increased neoepitope load, and higher PD-L1 expression may synergize to enhance response to immunotherapies (Fig. 6). APOBEC3s may also facilitate responsiveness to platinum-based therapies.

Number of clinical associations along with pre-clinical studies suggest that APOBEC3s could be used as biomarkers for currently available immunotherapies and chemotherapies, while novel startegies for APOBEC3 activation could prime certain tumors for increased therapeutic response (Fig. 6). In contrast, inhibiting APOBEC3s could slow tumor evolution, decrease subclone heterogeneity, and prevent therapy resistance in some cancers and in context to specific treatment types. APOBEC3s are dysregulated in a wide variety and high proportion of human tumors, so research in these areas holds great potential and could lead to new treatment options for many caner types.

## Data Availability

Not applicable.

## Abbreviations

APOBECs: Apolipoprotein B mRNA-editing enzyme, catalytic polypeptides
BKPyV: BK polyomavirus
TpC: Thiamine preceding cytosine
DDR: DNA damage response
ER: Estrogen receptor
HNSCC: Head and neck squamous cell carcinoma
HR: Homologous recombination
HPV: Human papilloma virus
IFN-γ: Interferon gamma
MDSCs: Myeloid-derived suppressor cells
NHEJ: Non-homologous end joining
TAMs: Tumor-associated macrophages
TNF-α: Tumor necrosis factor alpha
TLS: Translesion synthesis
ssDNA: Single-stranded DNA
SNP: Single-nucleotide polymorphism
